# Induction of autophagy mitigates TDP-43 pathology and translational repression of neurofilament mRNAs in mouse models of ALS/FTD

**DOI:** 10.1186/s13024-020-00420-5

**Published:** 2021-01-07

**Authors:** Sunny Kumar, Daniel Phaneuf, Pierre Cordeau, Hejer Boutej, Jasna Kriz, Jean-Pierre Julien

**Affiliations:** Department of Psychiatry and Neuroscience, CERVO Brain Research Centre, University Laval, 2601, Chemin de la Canardière, Quebec City, QC G1J 2G3 Canada

## Abstract

**Background:**

TDP-43 proteinopathy is a pathological hallmark of many neurodegenerative diseases including amyotrophic lateral sclerosis (ALS) and frontotemporal dementia (FTD). So far, there is no therapy available for these neurodegenerative diseases. In addition, the impact of TDP-43 proteinopathy on neuronal translational profile also remains unknown.

**Methods:**

Biochemical, immunohistology and assay-based studies were done with cell cultures and transgenic mice models. We also used Ribotag with microarray and proteomic analysis to determine the neuronal translational profile in the mice model of ALS/FTD.

**Results:**

Here, we report that oral administration of a novel analog (IMS-088) of withaferin-A, an antagonist of nuclear factor kappa-B (NF-ĸB) essential modulator (NEMO), induced autophagy and reduced TDP-43 proteinopathy in the brain and spinal cord of transgenic mice expressing human TDP-43 mutants, models of ALS/FTD. Treatment with IMS-088 ameliorated cognitive impairment, reduced gliosis in the brain of ALS/FTD mouse models. With the Ribotrap method, we investigated the impact of TDP-43 proteinopathy and IMS-088 treatment on the translation profile of neurons of one-year old hTDP-43^A315T^ mice. TDP-43 proteinopathy caused translational dysregulation of specific mRNAs including translational suppression of neurofilament mRNAs resulting in 3 to 4-fold decrease in levels type IV neurofilament proteins. Oral administration of IMS-088 rescued the translational defects associated with TDP-43 proteinopathy and restored the synthesis of neurofilament proteins, which are essential for axon integrity and synaptic function.

**Conclusions:**

Our study revealed that induction of autophagy reduces TDP-43 pathology and ameliorates the translational defect seen in mice models of ALS/FTD. Based on these results, we suggest IMS-088 and perhaps other inducers of autophagy should be considered as potential therapeutics for neurodegenerative disorders with TDP-43 proteinopathies.

**Supplementary Information:**

The online version contains supplementary material available at 10.1186/s13024-020-00420-5.

## Background

Cytoplasmic mislocalization and aggregation of TDP-43 are a pathological hallmark of many neurodegenerative diseases including ALS, FTD, limbic-predominant age-related TDP-43 encephalopathy (LATE) and Alzheimer’s disease [1–7]. TDP-43 is a DNA/RNA binding protein predominantly localized in the nucleus and it helps in RNA transcription, splicing, trafficking and chromatin condensation [1, 2]. In addition, TDP-43 also participates in translational regulation [8–10]. TDP-43 cytoplasmic increase has been shown to suppress global translation by binding with RACK1 on polyribosome [11]. However, the impact of TDP-43 proteinopathy on neuronal translational profile in vivo remains unknown. Transgenic mice expressing TDP-43 mutations have been described by different groups and many of them exhibit pathological changes reminiscent of human ALS and FTD [12, 13]. Different studies showed that clearance of excess cytoplasmic TDP-43 ameliorated the disease pathology in such mouse models of ALS [3, 14, 15]. Previously, our group reported that in context of ALS cases, TDP-43 can interact with the p65 NF-κB subunit to enhance its signalling activity in the central nervous system (CNS) [16]. Recently, we reported that AAV-mediated delivery of a single-chain antibody capable to block TDP-43 interaction with p65 NF-ĸB led to reduction of NF-ĸB activity and of TDP-43 aggregates in mouse models of ALS/FTD [3]. Reducing the NF-ĸB activity ameliorated motor function in mouse models of ALS [16, 17]. An extract of the medicinal plant *Withania somnifera* and its active compound Withaferin-A, an inhibitor of NF-ĸB signalling, conferred beneficial effects when administered in ALS/FTD mouse models [14, 16]. A recent study has also shown that withaferin-A protects the dopaminergic neurons and motor function in aging rats [18]. Withaferin-A was found to disrupt the NF-κB essential modulator (NEMO) reorganization into ubiquitin-based signaling structures by covalently modifying Cysteine-397, producing a lack of IKKβ activation [19].

In the present study, we have derived and tested the efficacy of oral administration of novel semi-synthetic analog of withaferin-A named IMS-088, in transgenic mice models of FTD/ALS expressing human TDP-43 mutants. IMS-088 reduces NF-ĸB signaling activity but is less toxic than withaferin-A. Treatment of mice expressing hTDP-43 mutants with IMS-088 ameliorated cognitive deficits, reduced cytoplasmic TDP-43 aggregates and enhanced levels of autophagy markers. We also studied by mass spectrometry of newly synthesized peptides of ribosomes the impact of IMS-088 treatment on neuronal translational profile with the use of double transgenic RiboTag;hTDP-43^A315T^ mice. We report for the first time that TDP-43 proteinopathy causes a translational dysregulation of selective mRNAs, including substantial repression of neurofilament mRNAs. IMS-088 treatment mitigated TDP-43 pathology and it restored neurofilament protein synthesis in ALS/FTD mice.

## Methods

### Cell culture

Mouse microglial (BV2) and motor neuron like (NSC-34) cells were stably transfected with pGL4.32[luc2P/NF-κB–RE/Hygro] plasmid DNA (Promega, Madison, WI, USA). The vector expressed 5 copies of NF-ĸB response element that drives transcription of the luciferase reporter gene luc2P. These cells were cultured in Dulbecco’s Modified Eagle Medium 16 (DMEM) supplemented with 10% fetal bovine serum. For clonal selection of the transfected plasmid these cells were cultured in 100 μg/ml hygromycin. HEK 293 cells were also used and was cultured in Dulbecco’s Modified Eagle Medium (DMEM) containing 10% fetal bovine serum. The culture media for all the cells, we added 100 units/ml penicillin-streptomycin.

### Evaluation of luciferase activity assay

To measure p65 luciferase activity, BV2 or NSC-34 cells (5 × 10^4^ per well) were seeded in 24-well plates. The BV2 or NSC-34 cells were stimulated with 500 ng/ml bacterial lipopolysaccharide (LPS) or 40 ng/ml of TNFα respectively. In BV2 cell experiment, after 2 h, the media was removed and new media containing LPS with or without IMS-088 or withaferin-A was added for next 2 h. Similarly, for NSC-34 cells after 4 h of TNFα incubation, media was replaced with media containing TNFα with IMS-088 or withaferin-A for 2 h. Post-treatment, the media was removed, and cells were gently washed with 1× PBS, and then lysed using Glo Lysis buffer (Promega, Madison). Luciferase activity was measured using the Bright-Glo Luciferase assay system (Promega,Madison), according to the manufacturer’s instructions. DMSO treated cells under similar conditions served as control. Results were expressed as mean of luciferase activity/μg of total protein from at least 3 wells in each treatment condition for BV2 cells and 4 wells of each treatment for NSC-34 cells.

### Evaluation of cell survival

BV2 and NSC-34 cells were seeded onto 24-well plates at a density of 5X10^4^ cells/well. The treatment paradigm was similar as explained for luciferase assay. Post incubation with the drug, cell viability was measured using MTS assay using [3-(4,5- dimethylthiazol-2-yl)-5-(3carboxymethoxyphenyl)-2-(4-sulfophenyl)-2H-tetrazolium],as per the manufacturer’s instructions (Promega, Madison). The absorbance of the formazan adduct formed was determined at 490 nm using an EnSpire 2300 Multilabel reader (Perkin Elmer, Waltham, MA, USA). Values were expressed as arithmetic measurement unit.

### Inhibition of Autophagosome/lysosome pathway

Bafilomycin A1 (Sigma-Aldrich, USA), an inhibitor of Autophagosome/Lysosome pathway was used to test the effects of autophagy inhibition on IMS-088-mediated reduction of TDP-43 aggregation. HEK 293 cells were treated for 3 h with 50 μM Ethacrynic acid (in serum free media). Then, Bafilomycin A1 (300 nM) with or without IMS-088(1 μM) was added to media for 6 h. After treatment, analysis of insoluble, or soluble hTDP-43 was done with 6 M urea and RIPA buffers respectively followed by immunoblotting.

### Sample preparation and immunocytochemistry

HEK-293 cells were treated with 50 μM of Ethacrynic acid (Millipore sigma) overnight with or without 1 μM IMS-088.Post-treatment, cells were washed with PBS, fixed in 4% PFA for 10 min followed by permeabilization with 0.1% Triton X containing PBS (PBST). After permeabilization cells were washed and blocked with 10% goat serum, followed by incubation in primary antibody against hTDP-43 (1:1000) at room temperature overnight. Next morning, the cells were washed with PBST and incubated with the fluorochrome-conjugated secondary antibody (1:500) for 1 h at room temperature in dark. After secondary antibody incubation, cells were washed and incubated in DAPI for 60 s. Following five washes cells were mounted and observed under a fluorescence microscope (Zeiss, Germany).

### Mouse models and drug treatment

Transgenic mice expressing hTDP-43A315T or hTDP-43G348C were used for the experiment. Randomly selected animals were used for IMS-088 or vehicle treatment groups. IMS-088 was developed and generously provided by IMSTAR therapeutics, Canada. The average age of the mice at the start of the treatment was 1 year (pathological stage). 30 mg/kg of body weight IMS-088 (by gavage) was given twice a day for 8 weeks. Animals in the vehicle group received equal volumes of buffered saline for the same durations. Post-treatment, we blindly split each group into different subgroups and used all the mice for further experiments (Additional file 5). The Animal Care Ethics Committee of Université Laval approved all in vivo experimental protocols. Experiments were carried out in accordance with the Guide for the Care and Use of Experimental Animals of the Canadian Council on Animal Care.

### Passive avoidance and novel object recognition test

For passive avoidance test, the mice were first conditioned in a light-dark chamber with getting foot-electric shock when they enter in the dark chamber. Next day, one-trial passive avoidance was performed as described earlier [20] to check whether they avoid entering the dark chamber based on their memory function. The latency time for mice to enter the dark compartment was measured for the final test, with a cut-off time of 300 s.

Novel object recognition is a 3-day test, as previously described [21], in a 20 × 50 × 30 cm Plexiglas box for 5 min per session. Briefly, on the first day of trial, the mice were kept it in the empty box to familiarize with the environment. On the second day, two similar objects were placed in the box for mice to familiarize with the objects. On the final day of test, one of the objects was replaced with a new object and then the time spent by mice on the new object was measured for the different groups to compare the cognitive performance.

### Immunohistochemistry and image analysis

Mice were anesthetized by intraperitoneal injection using 10 μl/gm pentobarbital 12 mg/ml. The animals were transcranial perfused with ice cold phosphate buffered saline and 4% Paraformaldehyde. Post-perfusion brains and spinal cords were excised and post-fixed overnight in 4% PFA at 4 °C. For cryoprotection brains and spinal cords were kept in 30% sucrose solution at 4 °C for a day and 25 μm sections were cut using a sliding VT 1200S vibratome (Leica Microsystems). Further the sections were mounted on the glass slides to procced with immunohistochemistry.

Section containing slides were washed three times with PBS (5 min) at room temperature and then antigen retrieval was performed for 20 min at 98 °C using 6 M sodium citrate buffer. Slides were washed with 1X PBS after to room temperature. The sections were then washed in 0.25% Triton X-100 in PBS (PBST) (3 X 5 min) and then blocked for 1 h in 10% goat serum in PBST in at room temperature. The sections were then incubated with primary antibodies overnight at room temperature. Next morning the sections were washed (3 × 10 min) with 0.25% Triton X-100 in PBS and incubated with desired secondary antibodies for 90 min at room temperature in a dark chamber. Prior to mounting sections were incubated with DAPI (1 min), treated with true black (1 min) and then washed (3 × 10 min) with 0.25% Triton X-100 in PBS. Mounting was done onto glass slides using mounting media.

Fluorescent signal was detected using LSM 700 inverted confocal microscope (Zeiss) or Apotome (Zeiss). For each experiment, the best acquisition parameter has established considering the sections are not overexposed and the saturated signals can be avoided.

For quantifying mean fluorescence intensities of iba1 signals, at least 5–7 images per mice were captured at 20X using z-stack imaging method with Zeiss confocal microscope. We considered hippocampus and cortex separately as region of interest for analysis. Further, the quantification was performed using ImageJ software with the maximum intensity projection image. The average values were compared between saline and drug treated group. The data was represented as mean + standard error of mean (sem.)

### DNA constructs, generation of transgenic mice and genotyping

The DNA construct used for the generation of the NFL-HA-mRFP1-RPL10a transgenic mice were prepared as following. First, HA-mRFP1 fragment was obtained by PCR using the following primers: 5′ primer: 5′-GGG ACG ACG AAT TCG GAG GCA GCA TGT ACC CAT ACG ATG TTC CAG ATT ACG CTG CCT CCT CCG AGG ACG T-3′ and 3′ primer: 5′-GGG ACG ACG GAT CCG GCG CCG GTG GAG TGG CGG CCC-3′.

Then, the amplified fragment was introduced into pBluescript KS+ plasmid into corresponding restriction sites. A 2.5 Kb BamHI /NotI fragment corresponding to the genomic DNA of 60s ribosomal protein L10a (RPL10a) was introduced into corresponding restriction sites of pBSKS-HA-mRFP1 recombinant vector. A 3.4 Kb. XhoI/XhoI fragment corresponding to the HA-mRFP1-mRPL10a transgene was introduced into pSKhNF-L plasmid instead of the exon 1. As described in Charron G. et al., 1995 [22], this plasmid contains human NF-L gene including − 292 bp of 5′ flanking sequences and intron sequences sufficient to drive NF-L expression in the nervous tissues of adult transgenic mice. To facilitate the digestion of the transgene, a KpnI restriction site was added to the pSKhNF-L plasmid at the position 6711 bp. The integrity of the final construct was verified by sequencing.

For microinjection, a KpnI-KpnI DNA fragment of 9.0 kb was isolated on agarose gel and purified using a QIAquick Gel Extraction Kit (Qiagen #28115). The transgenic mice NFL-HA-mRFP1-RPL10a named NFLrRFP were viable, did not develop overt phenotypes and were genotyped by PCR amplification. For genotyping, a 179 bp fragment from the mRFP1 gene is amplified from the NFLrRFP transgenic mice and not from the wild type mice. The PCR was performed on ear punch samples using the 5′ mRFP1-GEN primer: 5′-GACCGCCAAGCTGAAGGTGA-3′ and the 3′ mRFP1-GEN primer: 5′-CCGTCCTCGAAGTTCATCAC-3′.

The experiments presented in this paper were obtained by using the double transgenic mice named NFLrRFP;hTDP-43 ^A315T^ generated by crossing the NFLrRFP transgenic mice with our TDP43 ^A315T^ mice (supplementary figure 1B). All experimental procedures were approved by the Laval University animal care ethics committee and are in accordance with The Guide to the Care and Use of Experimental Animals of the Canadian Council on Animal Care.

### Protein extraction and immunoblotting

Both tissue and cell samples were lysed using RIPA buffer supplemented with protease inhibitor cocktail (Sigma, USA). Protein concentration was measured using Bradford reagent (Sigma, USA). 20–30 μg of protein samples were loaded and separated using SDS-PAGE followed by wet transfer on a methanol charged PVDF membrane. The membrane was blocked using 5% Bovine serum albumin for an hour at room temperature and incubated with primary antibodies overnight at 4 °C. The primary antibodies used at 1:1000 concentration were mouse monoclonal hTDP-43 (Abnova, Taiwan), LC3 (Novus Biologicals, USA), Beclin-1 (Novus Biologicals, USA), p62 (Millipore, USA), ATG-5 (Millipore, USA), and Actin (Millipore, USA), GFAP (Cell signalling technologies, USA),. Subsequently, the blots were incubated with either HRP-conjugated anti-rabbit or anti-mouse secondary antibodies. Moreover, the blots were developed using ECL detection reagents and visualized with a StarBright Blue 520 (Bio-Rad Laboratories, USA). All band intensities were quantified using the ImageJ lab software. The membranes were incubated with suitable peroxidase conjugated secondary antibodies (Vector Laboratories, USA). Once the incubation was over, PBST wash was given to the blots. Blots were then treated with ECL reagent and developed using UNITECH imaging system (Cambridge) from Millipore, CA USA.

### TRAP protocol

TRAP protocol described by Heiman and colleagues with small modifications was used for the study [23, 24]. Cortex and hippocampal tissue were extracted from the brain and homogenized (10% w/v) together in tissue lysis buffer. The samples were centrifuged at 2000 g for 10 min at 4 °C. Post-centrifugation, 1/9 sample volume of 10% NP-40 and 1/9 sample volume of 300 mM DHPC were added to the supernatant. Further the sample was gently mixed and then incubated for 30 min at 4 °C on orbital shaker. Post-incubation, centrifugation at 20,000 g for 10 min was given at 4 °C. The supernatant was further collected and divided into two equal volume parts (one part was used for mRNA isolation and the other for peptide extraction). Each sample was added to the anti-RFP agarose affinity resin and incubated overnight at 4 °C on orbital shaker. Next day, the beads were isolated using centrifugation and washed 4 times using high-salt buffer (20 mM HEPES-KOH [pH 7.3], 350 mM KCl, 12 mM MgCl2, 1% NP-40, 0.5 mM DTT, and 100 mg/mL cycloheximide. The beads pellet was used either for mRNA purification or peptide purification.

### Purification of mRNA after TRAP protocol

After the last washing, the beads were resuspended in 100 μL Nano prep lysis buffer with beta-mercaptoethanol for 10 min at room temperature. The RNA isolation was performed according to the kit manufacturer’s instructions (Absolutely RNA Nano prep kit). Three biological replicates were performed for each experiment (for each replicate, *n* = 2). Purified isolated RNA was subjected to Affymetrix mouse gene chip.

### Purification of peptides after TRAP protocol

At the end of washing, beads were resuspended in EDTA-elution buffer (10 mM HEPES-KOH [pH 7.3], 150 mM KCl, 5 mM MgCl2, 20 mM EDTA, and protease inhibitors) and incubated for 30 min at room temperature on orbital shaker. EDTA elution buffer was used to dissociate ribosomes and release nascent chain peptides. Eluate was recovered by centrifugation at 7000 rpm for 15 min. Collected ribosome associated peptides were sequenced by mass spectrometry using Orbitrap fusion mass spectrometer. Three biological replicates were performed for this experiment (*n* = 6 per condition).

### Statistical analysis

Prism 5.0 software (GraphPad, La Jolla, CA, USA) was used for all statistical analysis. Comparisons between 2 groups were done by unpaired two-tailed t test. Comparison between multiple groups was done by 1-way analysis of variance with Bonferroni’s or turkey post-test. A *p*-value up to 0.05 was considered significant.

## Results

### IMS-088 treatment inhibits NF-κB activation in vitro

**Fig. 1:**
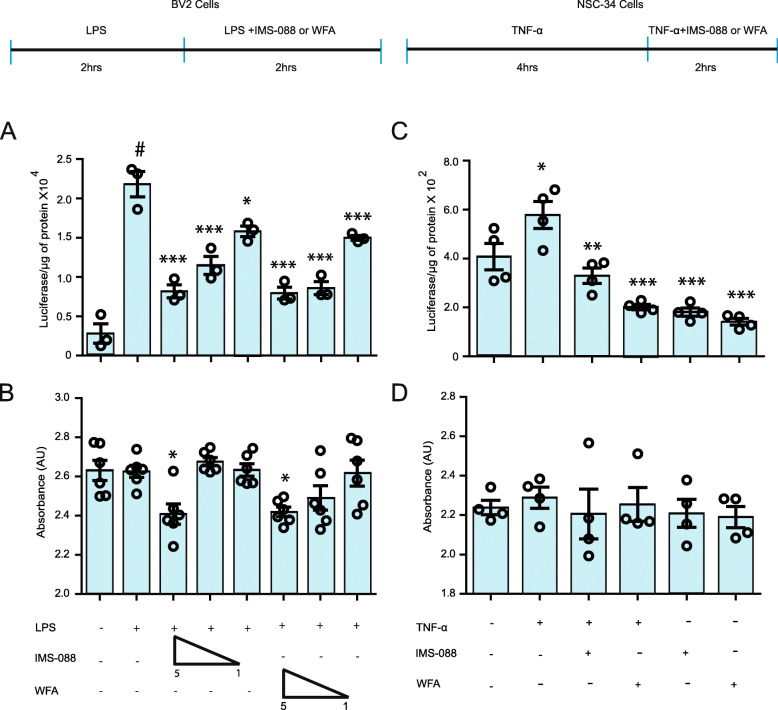
IMS-088 treatment Inhibits NF-κB activation in vitro*.* **a** Stably transfected BV2 cells with pGL4.32[luc2P/NF-κB–RE/Hygro] plasmid carrying 5 copies of an NF-κB response element that drives transcription of the luciferase reporter gene luc2P were used for the experiment. LPS treatment was given to these cells with bacterial LPS (500 ng/ml) for 2 h. Post-treatment of LPS stimulated cells were treated with varying concentrations of IMS-088 or WFA in LPS containing media for 2 h (*n* = 3; one way ANOVA; Tukey multiple comparison test). **b** Cell death assay seen in BV2 cell by addition of lipopolysaccharide (LPS), Withaferin A (WFA) or IMS-088 in dimethyl sulfoxide (DMSO) at varying concentrations (one way ANOVA; Bonferroni’s multiple comparison test; *n* = 6). **c** Stably transfected NSC-34 cells with pGL4.32[luc2P/NF-κB–RE/Hygro] plasmid carrying 5 copies of an NF-κB response element were used for the experiment. TNF-α (40 ng/ml) treatment was given to the cells to induced NF-κB luciferase activity. TNF-α stimulated cells were post-treated with 1 μM of IMS-088 or WFA in TNF-α containing media (*n* = 4, one way ANOVA; Tukey’s multiple comparison test). **d** Cell death difference observed in NSC-34 cell by addition of TNF-α, Withaferin A (WFA) or IMS-088 in dimethyl sulfoxide (DMSO) (*n* = 4; one way ANOVA; Bonferroni’s multiple comparison test). (#*P* < 0.0001 *** *p* < 0.0001, ** *p* < 0.001, * *p* < 0.05)

The NF-κB pathway is one of the major pathways which regulates neuroinflammation and can be activated by different inflammatory insults including bacterial lipopolysaccharide or TNF-α. To test the NF-κB inhibitory potential of IMS-088, we primed BV2 cells (microglial cell line) stably transfected with NF-κB-P65–luciferase reporter with LPS for 2 h after which the cells were exposed to varying concentrations of IMS-088 or WFA. We found significant 4-fold upregulation in NF-κB activity in LPS-treated group in comparison to DMSO treated group (Fig. 1a). Treatment with varying concentration of IMS-088 or WFA reduced the LPS-induced NF-κB activity in BV2 cells in a dose-dependent manner between 1 to 5 μM (Fig. 1a). Further to test the anti-NF-κB property of IMS-088 in neuronal cells, we treated NSC-34 cells stably transfected with NF-κB P65–luciferase reporter with TNFα (40 ng/ml) for 4 h. Then, the cells were exposed to IMS-088 or WFA (1 μM). There was a significant increase of 1.5-fold in NF-κB activity after TNF-α treatment whereas post-treatment with IMS-088 or WFA caused significant reduction of 3 folds in NF-κB activity (Fig. 1b). No cell loss was detected in any of the treatment groups except a 30% loss of BV2 cells treated with IMS-088 or WFA at 5 μM in LPS-containing media (Fig. 1b, d).

### IMS-088 treatment reduces ethacrynic acid-induced TDP-43 proteinopathy in cultured cells

**Fig. 2:**
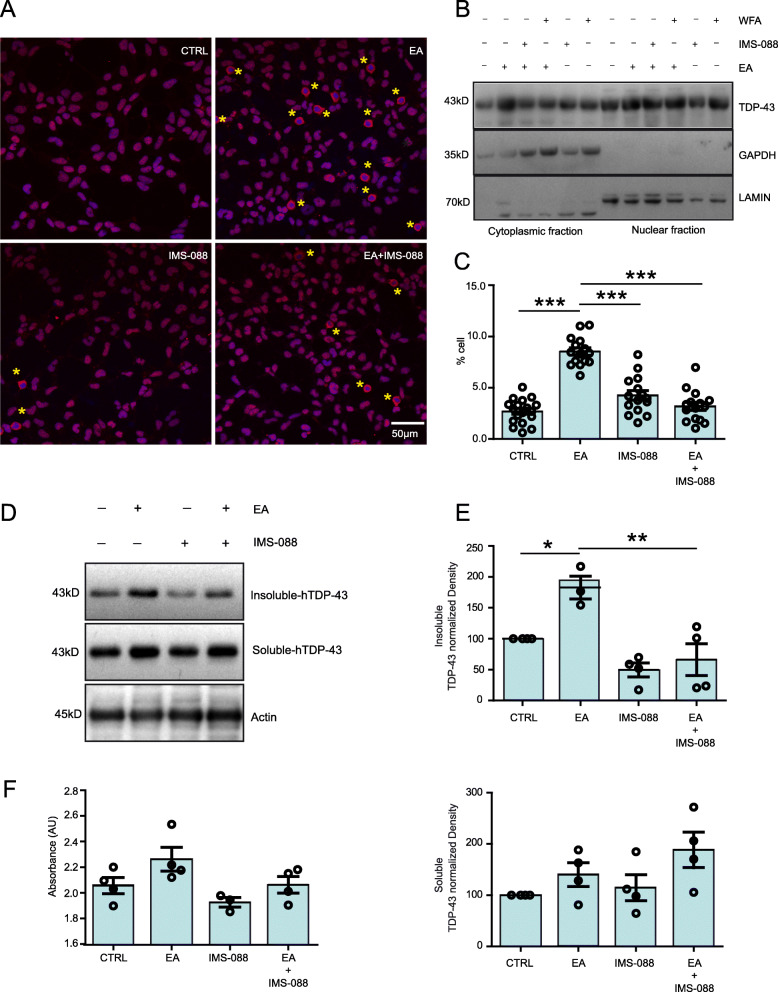
IMS-088 treatment reduces ethacrynic acid-induced TDP-43 proteinopathy in cultured cells. **a** Representative immunostaining and (**b**) immunoblot showing ethacrynic acid treatment induced cytoplasmic TDP-43 in HEK293 cells. Presence of IMS-088 in the media has reduced the cytoplasmic mis-localization in the cells. Pictures represent merge of hTDP-43 (red) and Hoechst (nuclei, blue) channels, Scale bar = 50 μm. **c** Data are represented as % cells showing TDP-43 cytoplasmic mis-localization [15] frames were analysed from 3 independent experiments). Data were analyzed by 1-way ANOVA with Bonferroni’s multiple comparison test as the post-test (***P < 0.0001). **d** Immunoblot and (**e**) quantification of RIPA soluble or insoluble TDP-43 in Hek293 cells received different treatments. (*n* = 3–4; 1-way ANOVA with Bonferroni’s multiple comparison test as the post-test). **f** Data showing cell survival assay difference observed in different treatment groups (*n* = 4; 1-way ANOVA with Bonferroni’s multiple comparison test as the post-test). Graphs show mean ± sem

It has been reported that ethacrynic acid (EA) treatment to cultured cells mimics the TDP-43 proteinopathy in vitro [25]. We used this paradigm to test in vitro therapeutic potential of IMS-088. We treated HEK293 cells overnight with 50 μM of EA in the presence or absence of IMS-088 (1 μM). Immunofluorescence microscopy and immunoblot revealed that treatment with EA significantly induced the cytoplasmic mis-localization of TDP-43 in comparison to the control DMSO- treated group. Remarkably, the presence of IMS-088 in the media resulted in a ~ 50% reduction of cells exhibiting cytoplasmic mis-localization of TDP-43 (Fig. 2a, b, c). Further, immunoblot of RIPA insoluble and soluble fractions revealed that the presence of IMS-088 in the media reduced the EA-induced aggregation of TDP-43 in cells (Fig. 2d, e). No cell loss was observed in any of the treatment groups (Fig. 2f).

### IMS-088 improved cognitive performance of transgenic mice expressing hTDP-43 mutants

IMS-088 passes the blood-brain barrier. A pharmacokinetic study in mice with ^14^C-IMS-088 given orally revealed that ^14^C-radioactivity loss from plasma occurred with a half-life of ~ 35 h while the radiolabel disappeared from the brain with a half-life of ~ 213 h*.*

**Fig. 3:**
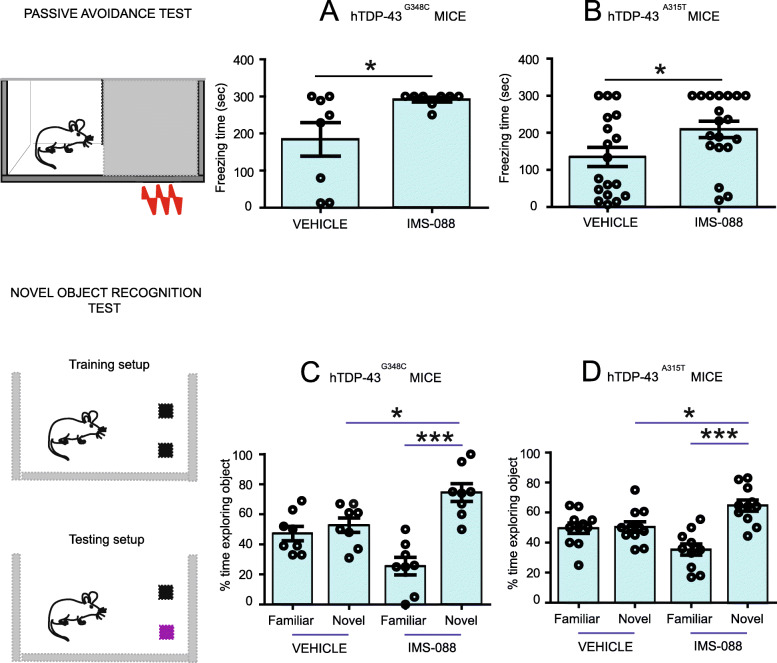
IMS-088 improved cognitive performance of transgenic mice expressing hTDP-43 mutants. Passive avoidance test at 12 months of age in (**a**) hTDP-43^G348C^ mice (vehicle vs IMS-088 treated groups) (**b**) hTDP-43^A315T^ mice (vehicle vs IMS-088 treated groups). Graphs show freezing time in second spent by mice. Novel object recognition test at 12 months of age in (**c**) hTDP-43^G348C^ mice (vehicle vs IMS-088 treated groups) (**d**) hTDP-43^A315T^ mice (vehicle vs IMS-088 treated groups). Graphs show percentage of time spent with novel objects, 1-way ANOVA with Bonferroni’s multiple comparison test as the post-test ** *p* < 0.01 and * *p* < 0.05

A dose of IMS-088 (30 mg/kg body weight) was administered orally (gavage) twice a day for 8 weeks to hTDP-43^A315T^ (*n* = 19) and hTDP-43^G348C^ (*n* = 8) transgenic mice staring at ~ 12 months of age. The control hTDP-43^A315T^ (*n* = 18) and hTDP-43^G348C^ (n = 8) mice received equal volumes of buffered saline. The treatment of IMS-088 or saline for 8 weeks had no effects on viability or body weight of animals. Post treatment, mice were subjected to passive avoidance and novel object recognition tests to assess cognitive functions. In the passive avoidance test, analysis revealed that the saline-treated hTDP-43^A315T^ and hTDP-43^G348C^ mouse often failed to recollect the foot shock during the training session with average latency to cross the dark chamber of 135.3 ± 25.80 s; *N* = 18 and 184.2 ± 45.01 s; *N* = 8 respectively. IMS-088 treatment significantly increased the latency of hTDP-43^A315T^ and hTDP-43^G348C^ mice to enter in the dark chamber with retention time between 209.3 ± 21.82 s; *N* = 19 and 291.3 ± 6.325 s; N = 8 respectively (Fig. 3a, b). For instance, majority of the IMS-088-treated hTDP-43^G348C^ mice (75%) scored 300 s whereas only 25% of the control hTDP-43^G348C^ mice scored 300 s. Further to assess temporal lobe-dependent memory function, the novel object recognition test was performed. During the training session, no difference was observed in behavior between the IMS-088-treated and the saline-treated groups. After the training session, we performed the choice phase test in which the IMS-088-treated mice expressing hTDP-43 mutants showed significant increase in the time spent with the novel object (65 to 70% time) in comparison to saline-treated control mice (45 to 50% time) (Fig. 3c, d).

### IMS-088 treatment reduced microglial and astrocyte activation in mutant hTDP-43 mice

**Fig. 4:**
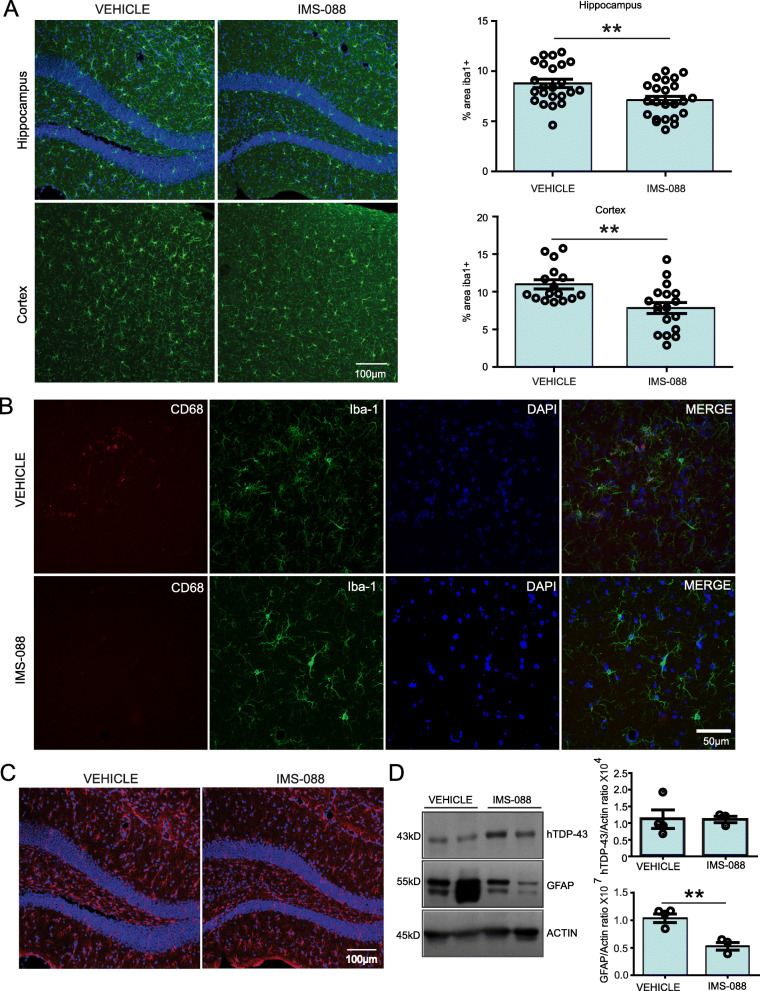
IMS-088 treatment reduced microglial and astrocyte activation in mutant hTDP-43 mice. **a** Representative immunostaining showing microglial cells in the cortex and hippocampal region of the hTDP-43 mutant mouse brains from the vehicle and IMS-088 treated groups. Graph presents quantification of % area covered by the iba1+ cells, using multiple sections from *n* = 3–4 mice, unpaired t-test; * *p* < 0.05. **b** Representative immunostaining of CD-68 + (red) and Iba1 (green) co-staining in vehicle and IMS-088 treated groups. **c** Representative image showing astrocytes in the hippocampal region of the hTDP-43 mutant mouse brains from the vehicle and IMS-088 treated groups. **d** Representing immunoblot and quantification of GFAP and total TDP-43 protein levels in the different treatment groups (*n* = 3–4). Graph represents the relative density of GFAP and total TDP-43 in the brain of vehicle and IMS-088 treated mice, unpaired t-test; ** *p* < 0.01

Since mutant hTDP-43 mice shows NF-κB induced inflammation, we tested anti-inflammatory properties of IMS-088. Immunofluorescence microscopy of brains sections was carried out using antibodies for detection of glial fibrillary acidic protein GFAP (astrocyte marker) and iba1 (microglial markers). As shown in Fig. 4a and b, IMS-088 treatment significantly reduced the immunostaining of iba1 protein in the microglia in cortex and hippocampus of the mice brain in compared to the saline-treated mice. Morphologically, more microglia were in reactive state occupying significantly larger area in the brain of vehicle-treated mice in comparison to IMS-088-treated mice. In IMS-088-treated group, the microglia exhibited small cell soma with distal arborisation in contrast to microglia in vehicle-treated mice which had large cell body with short thick processes.

In addition, immunoblot analysis revealed significant reduction in GFAP protein levels without changing the total TDP-43 levels in the IMS-088-treated group in comparison to vehicle-treated group. Consistent with reduction of astrocytosis, immunofluorescence revealed that morphologically the GFAP+ cells had thick processes and large cell body in vehicle-treated mice whereas the GFAP+ cells in the IMS-088- treated group were smaller (Fig. 4c, d).

### IMS-088 treatment reduced cytoplasmic TDP-43 aggregates

**Fig. 5:**
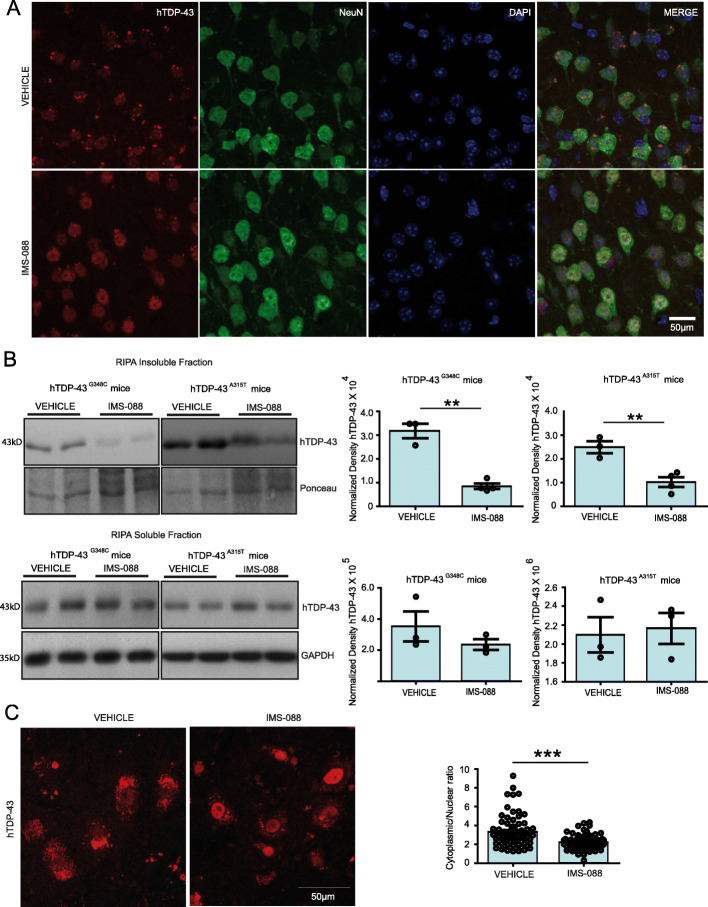
IMS-088 treatment reduced cytoplasmic TDP-43 aggregates in mutant hTDP-43 mice. **a** Representative pictures showing TDP-43 (red), NeuN (green) and DAPI (blue) in brain sections of vehicle and IMS-088 treated hTDP-43 mutant transgenic mice. **b** Representative immunoblots and quantification of RIPA insoluble and RIPA soluble fractions of brain from n = 3–4 independent mice from both hTDP-43^G348C^ and hTDP-43^A315T^ mice. The TDP-43 was normalized with ponceau or GAPD H. **c** Representative images and graph showing the cytoplasmic hTDP-43 in spinal motor neurons by IMS-088 treatment and the quantification of cytoplasmic to nuclear ration of hTDP-43 in hTDP-43^G348C^ mice (*n* = 75 neurons from 3 mice each group. Statistical analysis used was unpaired T-test. Data represents mean ± sem. ** *p* < 0.01; *** *p* < 0.001

During aging, hTDP-43^A315T^ and the hTDP-43^G348C^ transgenic mice develop cytoplasmic mis-localization and aggregation of TDP-43 in neurons [12]. We have examined the effect of 8-week treatment with IMS-088 on the distribution and aggregation of hTDP-43 in the brain cortex region. Immunofluorescence microscopy in (Fig. 5a) revealed abundant hTDP-43 aggregates in the cytoplasm of cortical NeuN+ cells in saline-treated hTDP-43^A315T^ mice at 1 year of age. Remarkably, treatment with IMS-088 restored the nuclear localization of TDP-43 and it substantially eliminated the presence of hTDP-43 aggregates. Consistent with the microscopy data, immunoblotting revealed decreases of 3 to 4 folds in levels of hTDP-43 recovered in the RIPA insoluble protein fractions of the brain (Fig. 5b). In contrast, the levels of soluble hTDP-43 in the brain did not differ significantly between IMS-088-treated and saline-treated mice expressing hTDP-43 mutants (Fig. 5b). Note that 35kD and 25kD fragments of TDP-43 were not detected in these experiments using the anti-human TDP-43 antibody. Interestingly, our data also revealed that in addition to insoluble level of hTDP-43, IMS-088 treatment has also significantly reduced the total level of phospho-TDP 43 by more than 2 folds in the brain of hTDP-43A315T mice (Fig. S1A).

Further, we measured the cytoplasmic to nuclear ratio in the large motor neurons of the spinal cord. Interestingly, treatment of IMS-088 has significantly reduced the cytoplasmic localization of TDP-43 in motor neurons of the hTDP-43^G348C^ mice (Fig. 5c). The combined data suggest that IMS-088 treatment has reduced TDP-43 proteinopathy in neurons.

### IMS-088 treatment boosted autophagy

**Fig. 6:**
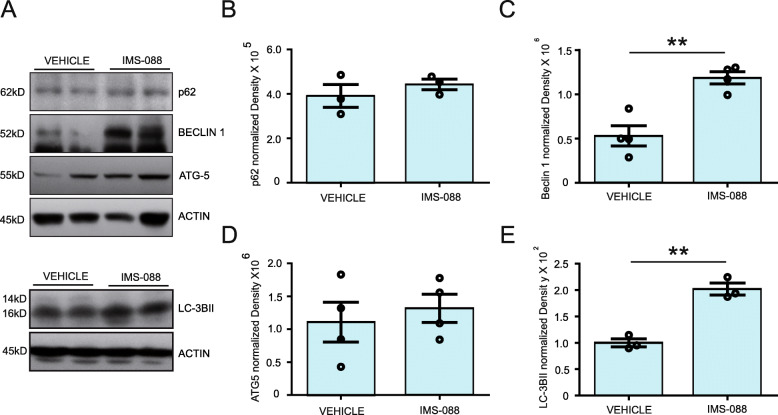
IMS-088 treatment boosted autophagy. **a** Representative immunoblots showing autophagic markers in the brain of hTDP-43^A315T^ mice received vehicle or IMS-088 treatment. Data Showing normalized density of (**b**) p62 (**c**) Beclin-1(**d**) ATG 5 (**e**) LC-3IIB in hTDP-43 mutant mice received either saline or IMS-088 (*n* = 3–4). Protein levels were normalized using Actin. Statistical analysis used was unpaired T-test. Data represents mean ± sem. ** *p* < 0.01

There is evidence suggesting that the inhibition of NF-κB signalling can potentially induce cellular autophagy [26]. Accordingly, we have investigated the effect of IMS-088 on levels of autophagic markers. After 8-week treatment with IMS-088, we detected a significant 2-fold increase in the levels of LC3BII, an autophagic marker, in comparison to vehicle-treated group (Fig. 6a, e). We also found an increase of 150% in Beclin-1, another autophagic marker, in the IMS-088-treated group (Fig. 6a, c). The levels of p62 and ATG5 remained unchanged (Fig. 6a, b, d). To further study IMS-088 mediated clearance of TDP-43 aggregates via autophagy, we blocked autophagosome-lysosome fusion using Bafilomycin A1 in HEK293 cells and analysed the levels of EA induced RIPA-insoluble and soluble TDP-43 levels. Immunoblot analysis has revealed a significant increased level of EA-induced RIPA insoluble-TDP-43 levels in IMS-088 with BafilomycinA1 treated group in comparison to IMS-088 alone (Fig. S2). We did not find any changes in RIPA-soluble level in any treated group (Fig. S2). From these results, we conclude that the IMS-088 treatment induced autophagic activity which may explain the clearance of cytoplasmic TDP-43 accumulations in brain and spinal neurons of TDP-43^A315T^ and TDP-43^G348C^ mutant mice.

### Translational suppression of neurofilament mRNAs in hTDP-43^A315T^ mice

Studies have shown that TDP-43 directly participates in regulation of translation [8–10]. TDP-43 proteinopathy in cells has also been shown to supress the global translation by interaction with RANK1 on polyribosome [11]. However, the impact of TDP-43 proteinopathy on neuronal translational profile in vivo remains unknown. Here, to investigate the molecular profiles of neuronal cells in vivo, we used a modified translational affinity purification (TRAP) and EDTA purified ribosomes associated nascent peptide chains (EDTA purified RANC) method [24]. This method involves the immunoprecipitation of the polyribosome complex with the attached mRNA and novel synthesizing peptides in a cell-specific manner from a complex tissue. To study the translational profile in neurons of the CNS, we have generated a transgenic mouse model named NFL-rRFP which expresses HA-RFP1 tagged rpl10 ribosomal protein under the human NEFL promotor (Additional file 5, Fig. S3). To address the effects of TDP-43 pathology on neuronal translational profile, the NFL-rRFP mice were bred with hTDP-43^A315T^ mice to derive NFL-rRFP; hTDP-43^A315T^ transgenic mice (Additional file 5, Fig. S3). The neuron-specific expression of RFP1 in NFL-rRFP; hTDP-43^A315T^ mice was confirmed by immunofluorescence microscopy of brain sections using NeuN as neuronal marker (Additional file 5, Fig. S3C).

**Fig. 7:**
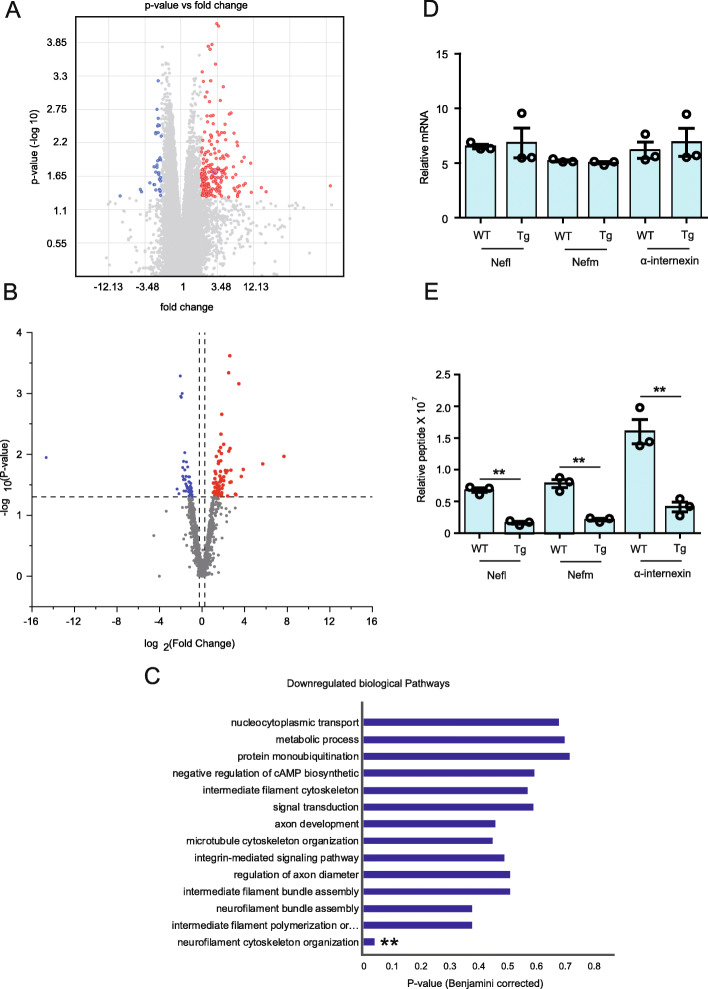
Translational suppression of neurofilament mRNAs in hTDP-43^A315T^ mice. **a** Volcano plot of Affymetrix 2.0 ST chip results of mRNA isolated from brain neuronal ribosome complex of NFL-rRFP and NFL-rRFP;hTDP-43^A315T^ mice at 12 months of age. Experiments were conducted in three biological replicates (*n* = 6 mice/condition). **b** Volcano plot of proteomic results of peptide isolated from brain neuronal ribosomal complex of NFL-rRFP and NFL-rRFP;hTDP-43^A315T^ mice analyzed with the mass spectroscopic methods at 12 months of age. Experiments were conducted in three biological replicates (*n* = 6 mice/condition). **c** GO functional analysis of isolated peptide from brain neurons of NFL-rRFP;hTDP-43^A315T^ mice revealed neuronal cytoskeletal deregulation. Data showing levels of ribosome-bound (**d**) mRNAs and (**e**) peptides for cytoskeletal proteins Nefl, Nefm and α-internexin in NFL-rRFP and NFL-rRFP;hTDP-43^A315T^ mice at 12 months of age. Data represents mean ± sem. ** *p* < 0.01

At 12 months of age when the NFL-rRFP;hTDP-43^A315T^ mice exhibited TDP-43 proteinopathy in cortical neurons, the brain tissue was collected for immunoprecipitation of ribosomes with anti-RFP antibody followed by purification and analyses of ribosome attached mRNA and newly synthesized peptides. The affymetrix Mouse Genome 430 analysis of mRNAs did not reveal significant changes for most of the genes. There was a differential regulation in only 0.13% mRNA bound to neuronal ribosomes in the NFL-rRFP;hTDP-43^A315T^ transgenic mice as compared to NFL-rRFP mice (Fig. 7a). Among the differentially regulated mRNAs, we found an upregulation of 80.23%mRNAs and a downregulation of 19.77% mRNAs in NF-L-RFP;TDP-43^A315T^ transgenic mice when compared to NFL-rRFP mice (Fig. 7a).

Further, mass spectroscopic analysis was performed with the EDTA-purified ribosomes associated nascent peptide chains isolated from ribosomal-complex immune-purification from brain neuronal cells. Interestingly, 117 different proteins exhibited significant changes in neuronal expression levels for NFL-rRFP;hTDP-43^A315T^ mice versus age-matched NFL-rRFP mice (Fig. 7b). Gene ontology (GO) functional analysis with upregulated peptides using David6.8 has revealed an upregulation in the metabolic pathways including mitochondrial function (Fig. S4A). In addition, levels of several peptides were highly increased in NFL-rRFP;hTDP-43^A315T^ mouse brain including Pin1 (peptidyl-prolyl cis-trans isomerase) which was approximately 5 folds higher than in samples from NFL-rRFP mice (Fig. S4D). Mitochondria-localized Pin1 has been reported to serve as a molecular switch that couples the phosphorylation of components of the apoptotic machinery to cell death processes, specifically in neurons.

Conversely, GO functional analysis with David software of the downregulated peptides revealed neuronal cytoskeletal disorganisation including a prominent down-regulation of neurofilament peptides in NFL-rRFP;hTDP-43^A315T^ mouse brain samples (Fig. 7c). Thus, levels of neurofilament peptides such as Nefl, NefM and α-internexin were decreased by 3.5 to 4 folds in NFL-rRFP;hTDP-43^A315T^ mice as compared to NFL-rRFP mice (Fig. 7e). Further, analysis of ribosome bound mRNA levels of Nefl, NefM and α-internexin has not shown any changes (Fig. 7d). The divergence of mRNA and protein levels data suggest that cytoplasmic TDP-43 accumulations in hTDP-43^A315T^ mice cause translational suppression of neurofilament protein synthesis. Note that other downregulated peptides were also significantly downregulated in hTDP-43^A315T^ mice brain neurons and their expression levels restored by IMS-088 treatment (Fig. S4D). For these other downregulated peptides in neuronal ribosomes of hTDP-43 mice there was no corresponding decreases in levels of ribosome-bound mRNA. This indicated that there was a translational blockade of these mRNA species.

### IMS-088 treatment reversed the neuronal translational defects associated with TDP-43 proteinopathy

**Fig. 8:**
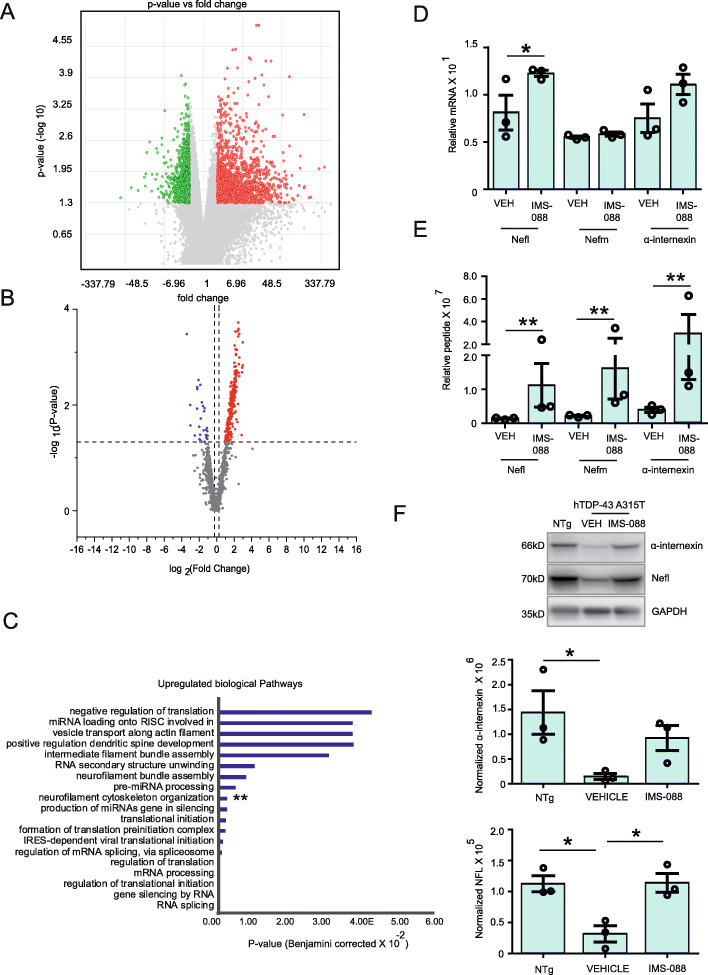
IMS-088 treatment reversed the neuronal translational defects associated with TDP-43 proteinopathy. **a** NFL-rRFP;hTDP-43^A315T^ mice treated with IMS-088 or vehicle for 8 weeks and the mRNA bound to neuronal ribosome complex were analyzed with the Affymetrix 2.0 ST chip at 12 months of age. **b** Volcano plot of proteomic results of peptide isolated from brain neuronal ribosomal complex of NFL-rRFP;hTDP-43^A315T^ mice treated with IMS-088 or vehicle were analyzed with the mass spectroscopic methods at 12 months of age. Experiments were conducted in three biological replicates (*n* = 6 mice/condition). **c** GO functional analysis of isolated peptide from brain neurons of NFL-rRFP;hTDP-43^A315T^ mice treated with IMS-088 or vehicle revealed IMS-088 rescued the neuronal cytoskeletal deregulation in mhTDP-43 mice. Levels of (**d**) mRNA and (**e**) peptide detected for neuronal cytoskeletal proteins nefl, nefm and α-internexin (Statistical analysis used was *p*-value limma test; Data represents mean ± sem. ** *p* < 0.01). **f** Immunoblot and graph represent levels of α-internexin and NFL in the mouse brain of different groups (*n* = 3; 1-way ANOVA; Bonferroni’s multiple comparison test, * *p* < 0.05)

To test the effects of IMS-088 on brain neuronal translational profiles, vehicle or IMS-088 (30 mg/kg) was administered twice-per-day for 8 weeks using gavage to NFL-rRFP;hTDP-43^A315T^ mice starting at approximately 10 months of age. In Affymetrix Mouse Genome 430 analysis, we found significant changes in only 4.13% of the mRNAs related to IMS-088 treatment (Fig. 8a). Among this 4.13% mRNAs, 56.09% mRNAs were upregulated whereas 43.91% mRNAs were downregulated in the upregulated mRNA group, 17.07% mRNAs were non-coding while in the downregulated mRNA group, 59.15% of genes were non-coding with unknown function (Additional file 5, Fig. S4C).

Mass spectroscopy analysis revealed that IMS-088 treatment of NFL-rRFP; hTDP-43^A315T^ mice significantly altered the synthesis of 199 different neuronal proteins (Fig. 8b). Of particular interest was the finding that IMS-088 treatment reversed the translational block of neurofilament mRNAs (Fig. 8c, e). Thus, the most prominent upregulation changes due to IMS-088 treatment of NFL-rRFP; hTDP-43^A315T^ mice were the 6 to 8-fold increase in neurofilament peptides such as Nefl, Nefm and α-internexin (Fig. 8e). The restoration of neurofilament protein synthesis by IMS-088 treatment in NFL-rRFP; hTDP-43^A315T^ mice was further confirmed by immunoblot analysis (Fig. 8f). In addition to neurofilaments, IMS-088 restored expression levels of specific peptides related to mRNA splicing, neurite outgrowth or apoptosis. As shown in Supplementary Fig. S4D, IMS-088 restored the levels of most peptides whose levels were deregulated in hTDP-43A315T mice. For instance, the levels of Pin1, srsf3 and camk4 were restored by IMS-088 treatment (Fig. S4D). Interestingly, apoptotic protein Pin1, which is upregulated in NFL-rRFP; hTDP-43^A315T^ mice was downregulated by 7.5 folds after IMS-088 treatment (Fig. S4D).

These mRNA and proteomic data provided evidence for neuroprotective effects of IMS-088. A most remarkable outcome of IMS-088 treatment was the restoration of synthesis of neurofilament proteins which were subjected to translational suppression in context of cytoplasmic TDP-43 mis-accumulation in hTDP-43^A315T^mice.

## Discussion

TDP-43 aggregates in motor neurons are found in the majority of ALS patients [27]. TDP-43 aggregates are also found in other neurodegenerative diseases including LATE [28] and 50% patients of FTD and Alzheimer’s disease [6, 7]. TDP-43 protein is involved in the regulation of RNA processing and splicing [1] as well as in chromatin condensation [2]. However, the impact of cytoplasmic TDP-43 mis-accumulation on mRNA translation in vivo has remained unknown. Here we used a Ribotag method [24] in combination with mass spectrometry to study the translational profiles of neurons in one-year old transgenic mice expressing ALS-linked hTDP-43^A315T^ mutant, a model of ALS/FTD [12]. Our study revealed for the first time that TDP-43 proteinopathy can cause translational deregulation of specific mRNAs. Remarkably, GO functional analysis indicated substantial suppression of mRNA translation for neurofilament Nefl, Nefm and α-internexin in hTDP-43^A315T^ mice. Neurofilaments are major cytoskeletal components in neurons. Transgenic mouse studies have highlighted the importance of neurofilament protein stoichiometry for correct assembly and transport [29–32]. Neurofilament disorganization can contribute to neuronal dysfunction and death [30, 33]. Reduced expression of Nefl or Nefm proteins has been shown to cause defect in synaptic function with ensuing behavioral impairment [34, 35]. Previous reports showed that TDP-43 can bind and stabilize neurofilament mRNAs [2, 36]. Here, our results suggest that cytoplasmic accumulations of TDP-43 can result in a gain of toxic function involving deregulation of mRNA translation and especially suppression of neurofilament protein synthesis. Our data did not reveal any changes in the levels of ribosome-bound mRNA of Nefl, Nefm, and alfa-internexin but they showed reduced levels of novel synthesizing peptides for these genes, suggesting translational blockage (Fig. 7d, e).

We report here the therapeutic effects of a novel withaferin-A analog, called IMS-088, in transgenic mice expressing hTDP-43 mutants. Withaferin-A, a compound extracted from the medicinal plant *Withania somnifera* has emerged with a therapeutic potential as inhibitor of NF-κB signalling pathway via NEMO interaction [19] and as inducer of autophagy [37]. Withaferin-A treatment conferred protection in two mouse models of ALS [16, 38]. Furthermore, .withaferin-A treatment of a mouse model of cerebral ischemia led to amelioration of pathological changes with reduction in NF-κB-mediated inflammation [39]. Despite beneficial properties of withaferin-A, this compound can induce cell death at high dose or prolonged treatment [40]. Addition of methoxy group to withaferin A significantly reduced toxic properties [41, 42]. Here, the IMS-088 compound obtained from IMSTAR therapeutics (Vancouver) is basically the 4-O-methyl withaferin-A which is better tolerated at high doses (60 mg/kg) than withaferin-A in mice. A pharmacokinetic study with ^14^C-labeled-IMS-088 revealed that this novel analog penetrates the blood brain barrier after oral intake. The present study shows that oral administration of IMS-088 for 8 weeks in transgenic mouse models with TDP-43 proteinopathy led to reduction of RIPA-buffer insoluble TDP-43 levels and of cytoplasmic TDP-43 aggregates in the brain and spinal cord. The IMS-088 treatment increased the levels of Beclin-1 and LC3BII which are autophagic markers. From these results, we conclude that clearance of cytoplasmic TDP-43 by IMS-088 in the mouse models is likely the result of a boost of autophagic activity. In other mouse models of neurodegenerative diseases, autophagy induction was also found to promote the clearance of different aggregated proteins including huntingtin [43], α-synuclein [44] and amyloid beta [45]. It has been suggested that autophagy insufficiency occurring during aging may contribute to increase of misfolded proteins [46].

Our data suggests that clearance of excess cytoplasmic TDP-43 by IMS-088 administration had beneficial effects in part by reversing the translational defects in neurons. Thus, IMS-088 treatment was found to restore the dysregulated translational profile of brain neurons in one-year old mice expressing hTDP-43 mutant. Of particular interest was the finding that IMS-088 rescued the translational repression of neurofilament proteins in the brain of the mice expressing mutant hTDP-43.

Treatment of mice expressing mutant hTDP-43 with IMS-088, an inhibitor of NF-κB activity, led to reduction of microgliosis and astrogliosis (Fig. 4). NF-κB is a transcription factor playing a key role in inflammatory responses including genes encoding cytokines and chemokines [47]. Microglia-specific inhibition of NF-κB pathway has also shown to attenuate neuroinflammation and extended survival by several weeks in the SOD1^G93A^ mice [17].

## Conclusions

We discovered with the use of transgenic mice expressing hTDP-43 mutants that cytoplasmic accumulation of TDP-43 can alter the translation of specific mRNAs in brain neurons including a suppression of neurofilament protein synthesis. Oral administration of IMS-088 reversed the translational damage in neurons, reduced inflammation and mitigated cognitive deficits. IMS-088 is a NF-κB inhibitor that induced concurrently autophagy, a process which likely contributed to clearance of cytoplasmic TDP-43 aggregates. The results suggest that IMS-088 and perhaps other inducers of autophagy might represent promising therapeutics for TDP-43 proteinopathies.

## Supplementary Information

**Additional file 1: Figure S1.** IMS-088 reduced levels of phospho-TDP43 in the brain of hTDP-43^A315T^ mice. The anti-phospho-TDP-43 antibody (pSer410, Sigma-Aldrich, USA) was used for immunodetection (*n* = 3 independent experiments).

**Additional file 2: Figure S2.** Inhibition of autophagy blocks IMS-088-mediated reduction of hTDP-43 aggregates. Immunoblots ad quantification of RIPA insoluble (A) and soluble (B) TDP-43 in HEK293 cells treated with ethacrynic acid with or without IMS-088 and Bafilmycin A1. HEK 293 cells were treated for 3 h with 50 μM Ethacrynic acid in serum free media and then with Bafilomycin A1 (300 nM) with or without IMS-088 for 6 h. Post-treatment, levels of RIPA insoluble (A) or soluble (B) hTDP-43 in Hek293 cells were determined by immunoblotting with anti-human TDP-43 antibody (Abnova). (*n* = 3; 1-way ANOVA with Bonferroni’s multiple comparison test as the post-test). Graphs show mean ± sem.

**Additional file 3: Figure S3.** (A) Schematic representation of HA-mRFP1-tagged murine Rpl10a ribosomal protein construct under control of the NFL 848 promoter to make neuron specific expression (B) Representative sketch showing the process used for generating double transgenic mice (by breeding NFL-RFP1 mice with mhTDP-43^A315T^ mice) (C) Representative image showing DAPI (blue), NeuN a neuronal marker and RFP (red) in the brain of hTDP-43^A315T^ mice and NfL-RFP;hTDP-43^A315T^ double transgenic mice. (D) Representative image of experimental protocol used for the Ribotrap experiment.

**Additional file 4: Figure S4.** (A) Representative data of upregulated peptide associated GO biological function in NFLrRFP;hTDP-43^A315T^ mice in comparison to NFLrRFP mice (B**)** Representative data of upregulated peptide associated GO biological function in NFLrRFP;hTDP-43^A315T^ mice treated with IMS-088 in comparison to saline treatment. (C) Pie chart showing the majority of mRNA dysregulated after IMS-088 treatment found in Affymetrix Mouse Genome 430 analysis were pseudo or non-regulated genes with unknown function (D) Representative data of altered peptide including Pin1 in NFL-RFP; hTDP-43A315T and the impact of IMS-088 treatment on the reversal of neuronal translational profile in NFL-RFP; hTDP-43A315T mice.

**Additional file 5.**
