# Supplementary figures and images for: Induction of autophagy mitigates TDP-43 pathology and translational repression of neurofilament mRNAs in mouse models of ALS/FTD
Source: Mol Neurodegener. 2021 Jan 7;16:1. doi: 10.1186/s13024-020-00420-5 (PMC7792109; doi:10.1186/s13024-020-00420-5)

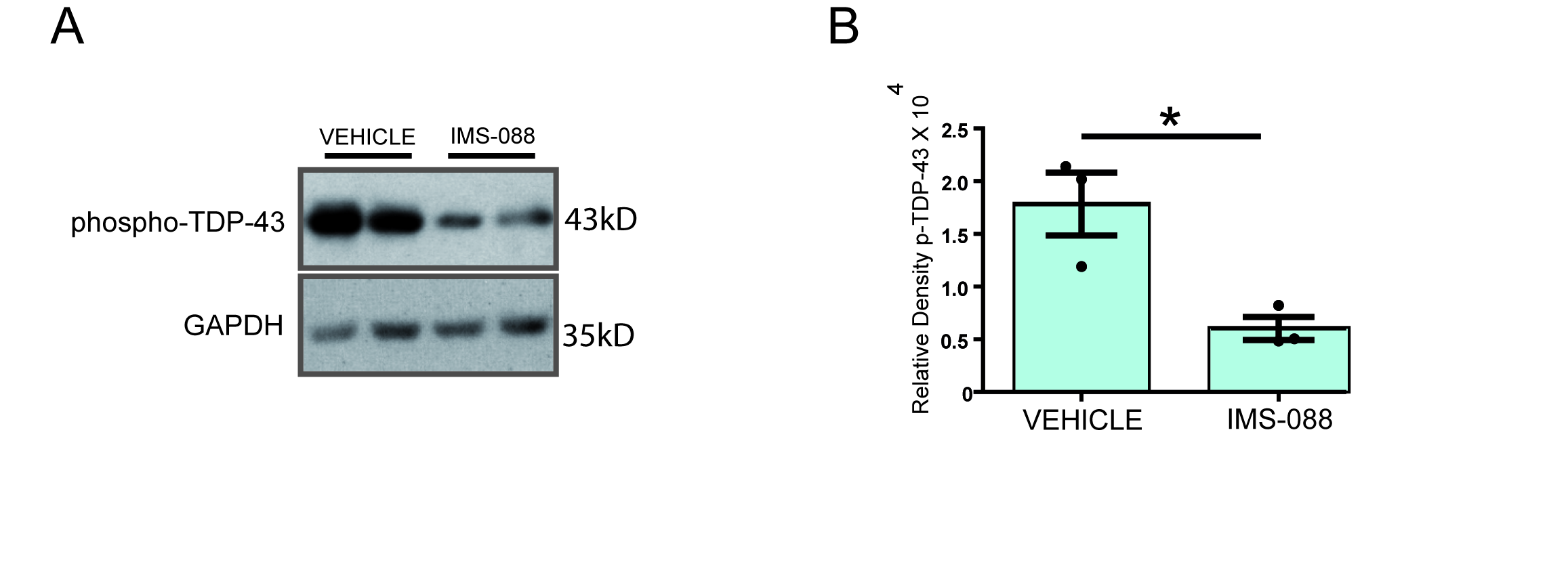

Supplement: Supplementary file 1 — Additional file 1: Figure S1. IMS-088 reduced levels of phospho-TDP43 in the brain of hTDP-43A315T mice. The anti-phospho-TDP-43 antibody (pSer410, Sigma-Aldrich, USA) was used for immunodetection (n = 3 independent experiments). [file 13024_2020_420_MOESM1_ESM.tif]

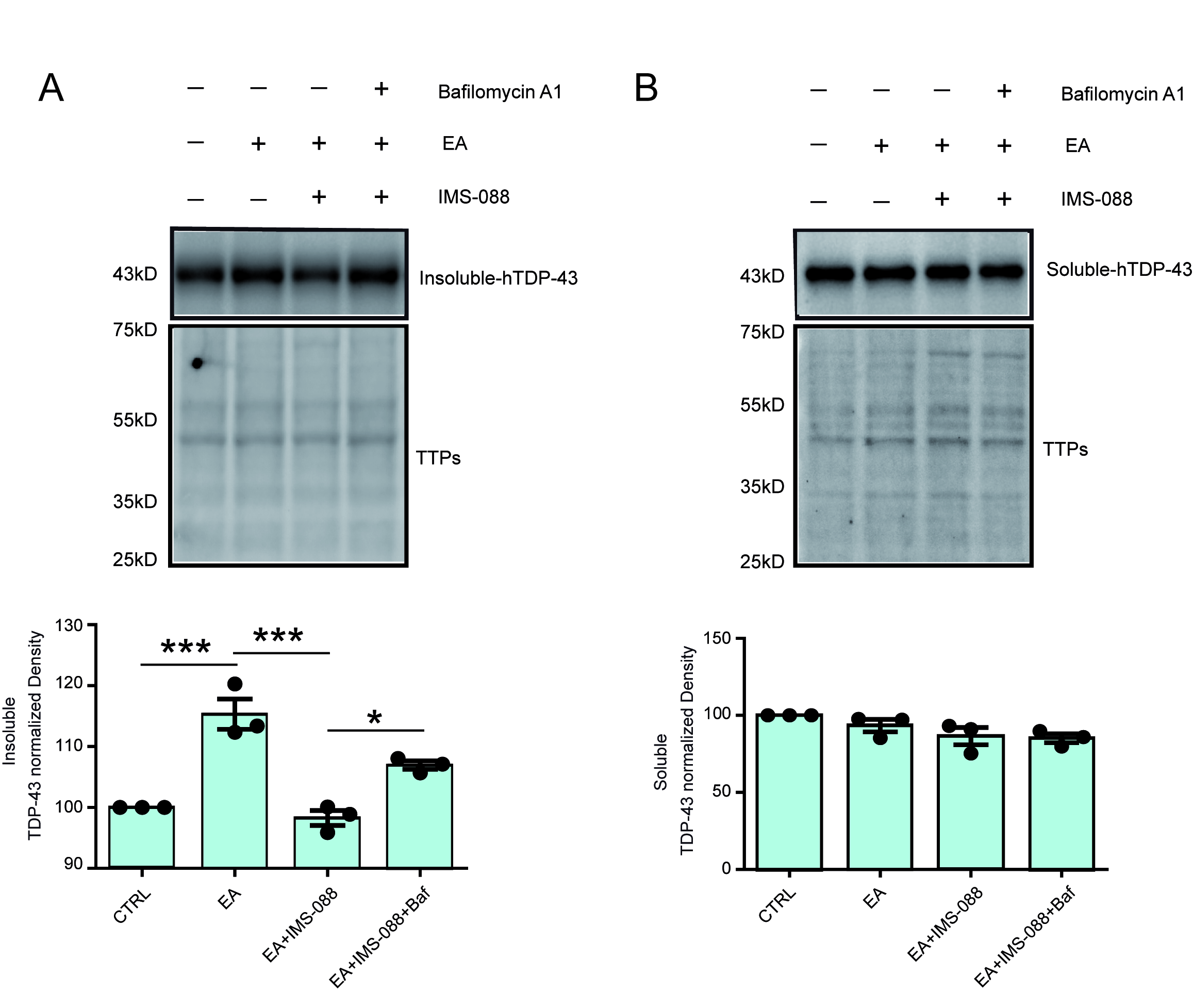

Supplement: Supplementary file 2 — Additional file 2: Figure S2. Inhibition of autophagy blocks IMS-088-mediated reduction of hTDP-43 aggregates. Immunoblots ad quantification of RIPA insoluble (A) and soluble (B) TDP-43 in HEK293 cells treated with ethacrynic acid with or without IMS-088 and Bafilmycin A1. HEK 293 cells were treated for 3 h with 50 μM Ethacrynic acid in serum free media and then with Bafilomycin A1 (300 nM) with or without IMS-088 for 6 h. Post-treatment, levels of RIPA insoluble (A) or soluble (B) hTDP-43 in Hek293 cells were determined by immunoblotting with anti-human TDP-43 antibody (Abnova). (n = 3; 1-way ANOVA with Bonferroni’s multiple comparison test as the post-test). Graphs show mean ± sem. [file 13024_2020_420_MOESM2_ESM.tif]

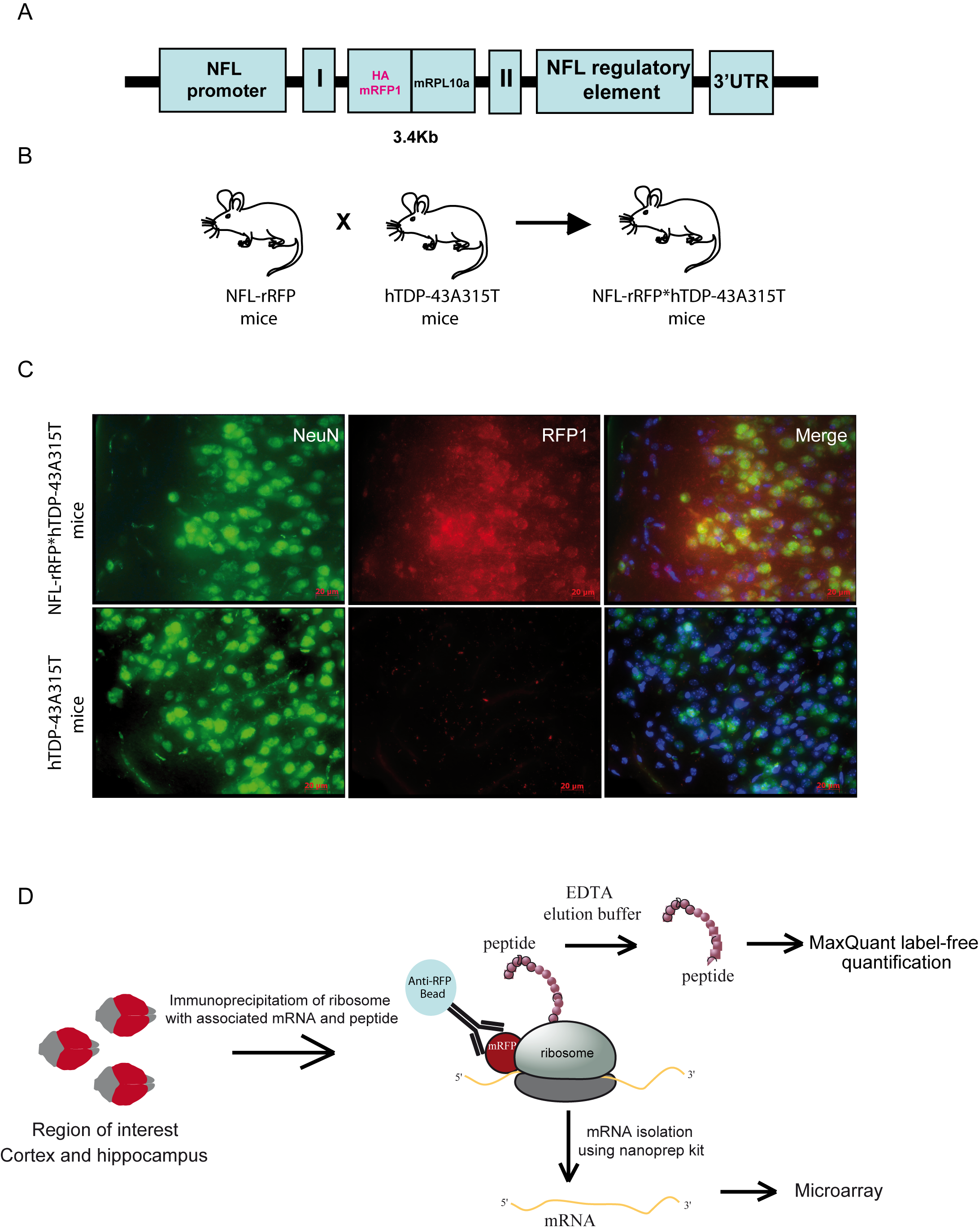

Supplement: Supplementary file 3 — Additional file 3: Figure S3. (A) Schematic representation of HA-mRFP1-tagged murine Rpl10a ribosomal protein construct under control of the NFL 848 promoter to make neuron specific expression (B) Representative sketch showing the process used for generating double transgenic mice (by breeding NFL-RFP1 mice with mhTDP-43A315T mice) (C) Representative image showing DAPI (blue), NeuN a neuronal marker and RFP (red) in the brain of hTDP-43A315T mice and NfL-RFP;hTDP-43A315T double transgenic mice. (D) Representative image of experimental protocol used for the Ribotrap experiment. [file 13024_2020_420_MOESM3_ESM.tif]

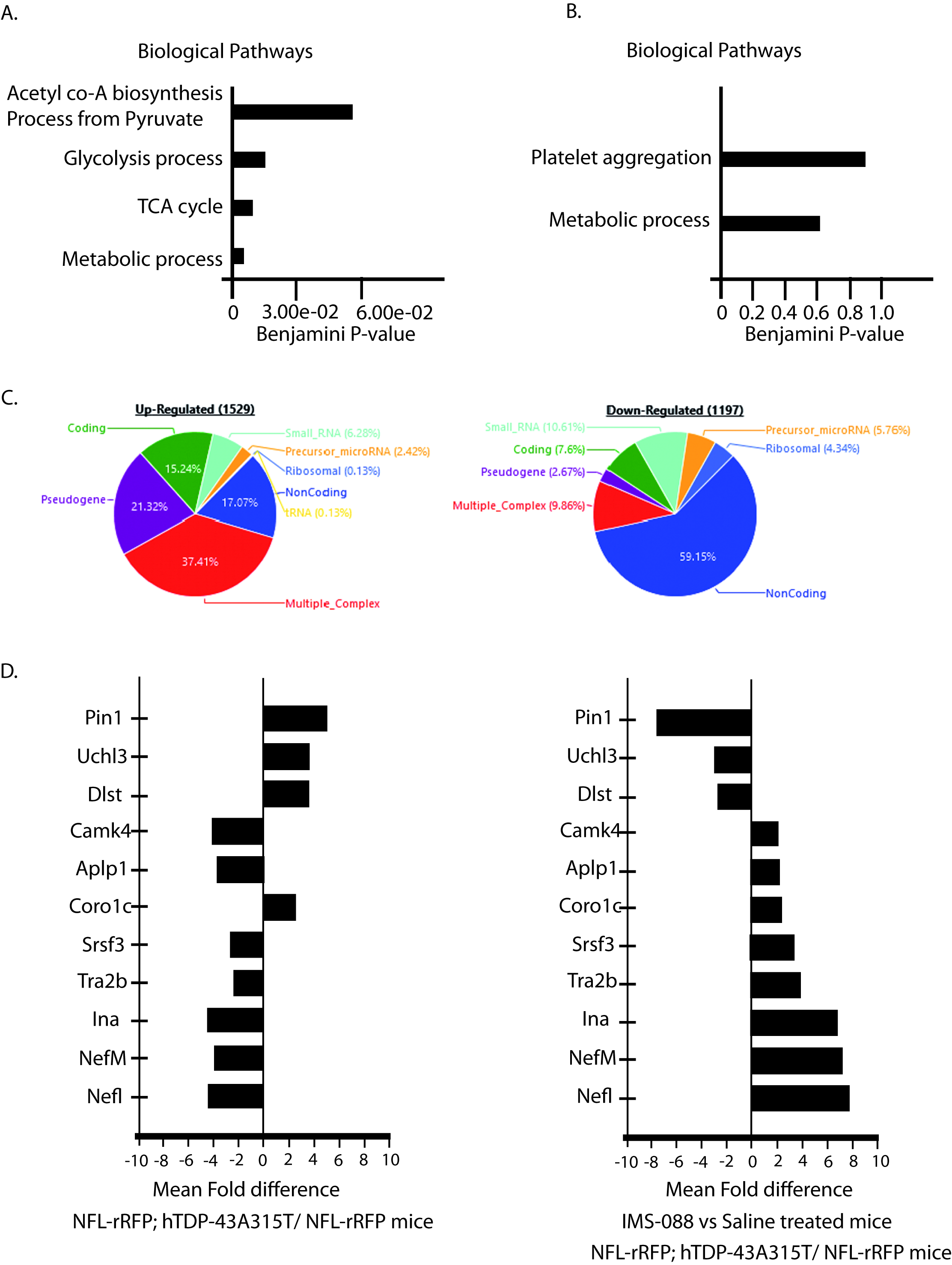

Supplement: Supplementary file 4 — Additional file 4: Figure S4. (A) Representative data of upregulated peptide associated GO biological function in NFLrRFP;hTDP-43A315T mice in comparison to NFLrRFP mice (B) Representative data of upregulated peptide associated GO biological function in NFLrRFP;hTDP-43A315T mice treated with IMS-088 in comparison to saline treatment. (C) Pie chart showing the majority of mRNA dysregulated after IMS-088 treatment found in Affymetrix Mouse Genome 430 analysis were pseudo or non-regulated genes with unknown function (D) Representative data of altered peptide including Pin1 in NFL-RFP; hTDP-43A315T and the impact of IMS-088 treatment on the reversal of neuronal translational profile in NFL-RFP; hTDP-43A315T mice. [file 13024_2020_420_MOESM4_ESM.tif]

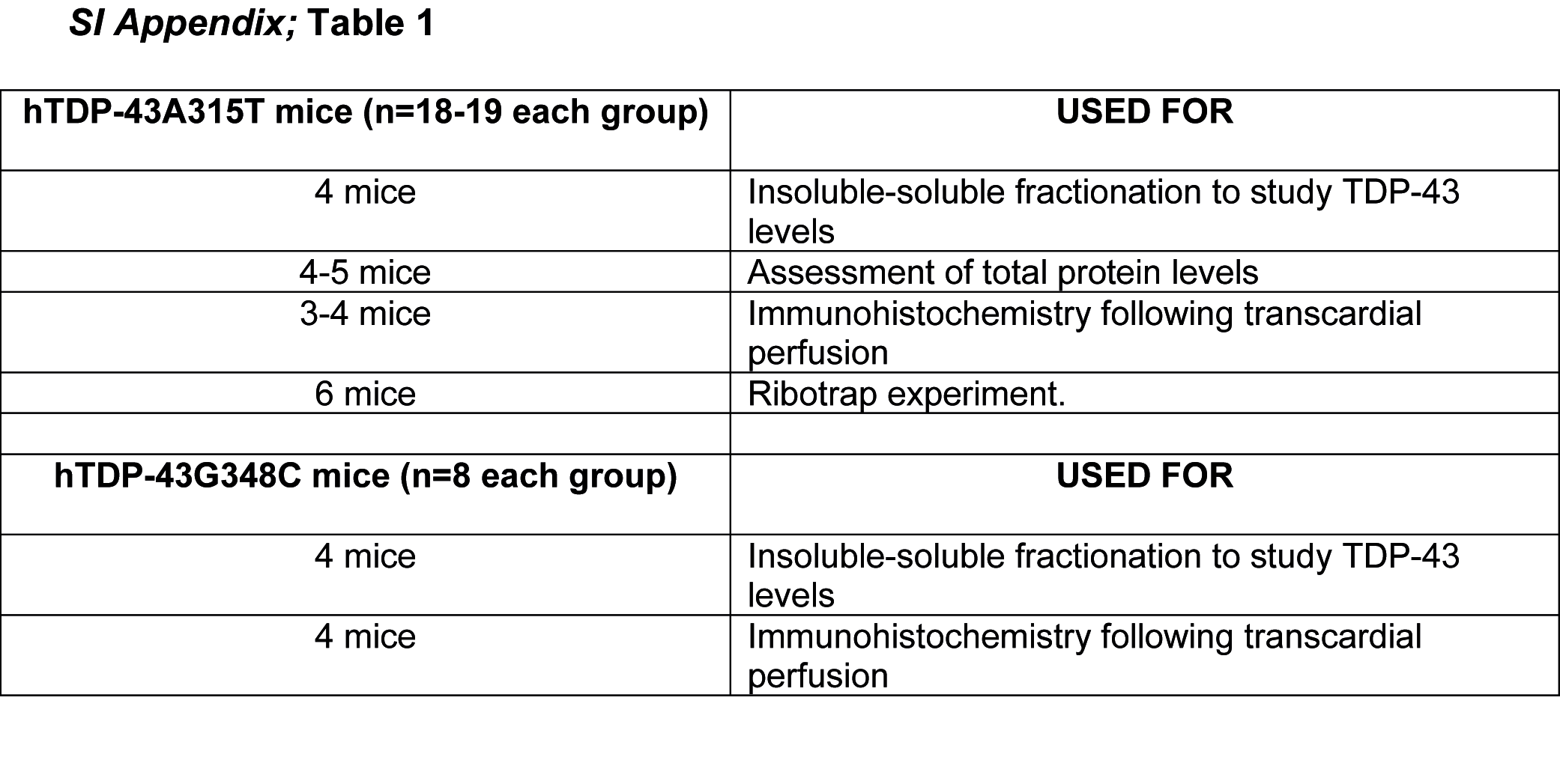

Supplement: Supplementary file 5 — Additional file 5. [file 13024_2020_420_MOESM5_ESM.tif]
